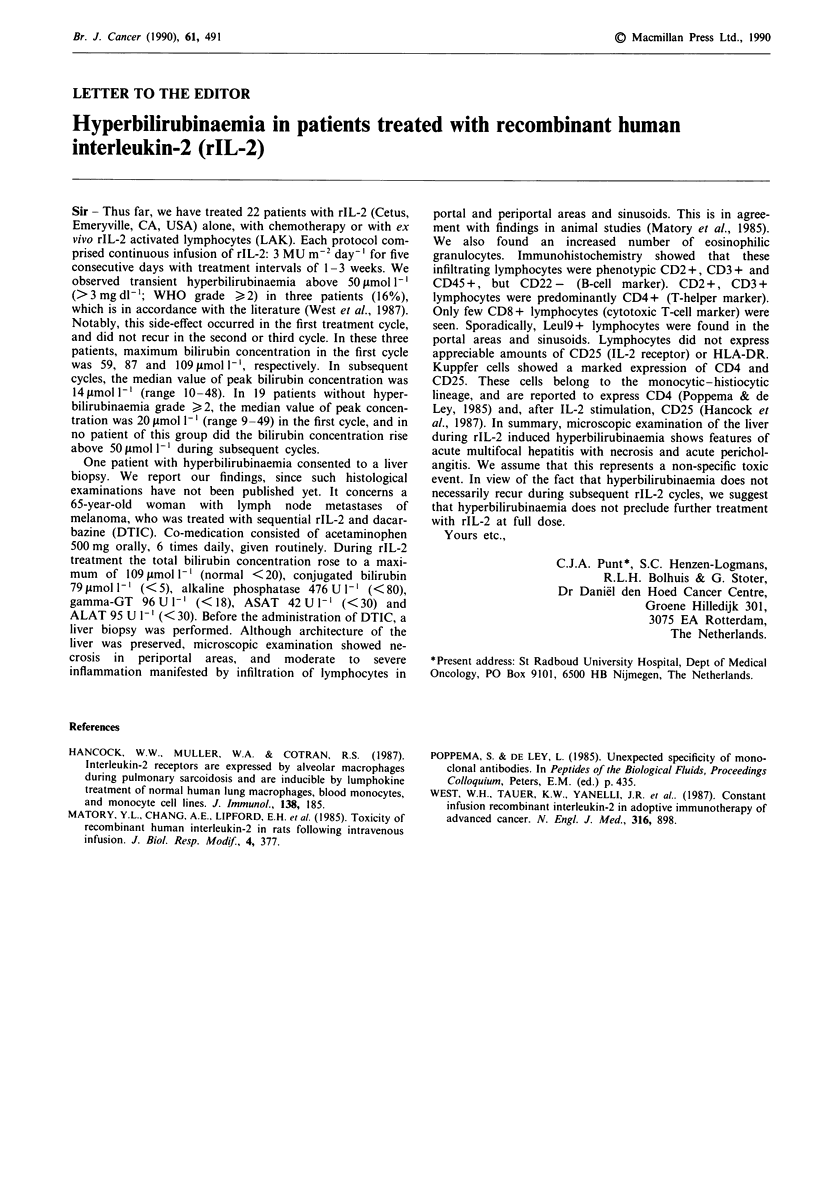# Hyperbilirubinaemia in patients treated with recombinant human interleukin-2 (rIL-2)

**DOI:** 10.1038/bjc.1990.108

**Published:** 1990-03

**Authors:** C. J. Punt, S. C. Henzen-Logmans, R. L. Bolhuis, G. Stoter


Br. J. Cancer (1990), 61, 491 © Macmillan Press Ltd., 1990

### LETTER TO THE EDITOR

### Hyperbilirubinaemia in patients treated with recombinant human interleukin-2 (rIL-2)

**Sir** – Thus far, we have treated 22 patients with rIL-2 (Cetus, Emeryville, CA, USA) alone, with chemotherapy or with *ex vivo* rIL-2 activated lymphocytes (LAK). Each protocol comprised continuous infusion of rIL-2: 3 MU $m^{-2}$ $day^{-1}$ for five consecutive days with treatment intervals of 1–3 weeks. We observed transient hyperbilirubinaemia above 50 $\mu mol\ l^{-1}$ ($>3\ mg\ dl^{-1}$; WHO grade $\geqslant 2$) in three patients (16%), which is in accordance with the literature (West *et al.*, 1987). Notably, this side-effect occurred in the first treatment cycle, and did not recur in the second or third cycle. In these three patients, maximum bilirubin concentration in the first cycle was 59, 87 and 109 $\mu mol\ l^{-1}$, respectively. In subsequent cycles, the median value of peak bilirubin concentration was 14 $\mu mol\ l^{-1}$ (range 10–48). In 19 patients without hyperbilirubinaemia grade $\geqslant 2$, the median value of peak concentration was 20 $\mu mol\ l^{-1}$ (range 9–49) in the first cycle, and in no patient of this group did the bilirubin concentration rise above 50 $\mu mol\ l^{-1}$ during subsequent cycles.

One patient with hyperbilirubinaemia consented to a liver biopsy. We report our findings, since such histological examinations have not been published yet. It concerns a 65-year-old woman with lymph node metastases of melanoma, who was treated with sequential rIL-2 and dacarbazine (DTIC). Co-medication consisted of acetaminophen 500 mg orally, 6 times daily, given routinely. During rIL-2 treatment the total bilirubin concentration rose to a maximum of 109 $\mu mol\ l^{-1}$ (normal $<20$), conjugated bilirubin 79 $\mu mol\ l^{-1}$ ($<5$), alkaline phosphatase 476 U $l^{-1}$ ($<80$), gamma-GT 96 U $l^{-1}$ ($<18$), ASAT 42 U $l^{-1}$ ($<30$) and ALAT 95 U $l^{-1}$ ($<30$). Before the administration of DTIC, a liver biopsy was performed. Although architecture of the liver was preserved, microscopic examination showed necrosis in periportal areas, and moderate to severe inflammation manifested by infiltration of lymphocytes in portal and periportal areas and sinusoids. This is in agreement with findings in animal studies (Matory *et al.*, 1985). We also found an increased number of eosinophilic granulocytes. Immunohistochemistry showed that these infiltrating lymphocytes were phenotypic CD2+, CD3+ and CD45+, but CD22− (B-cell marker). CD2+, CD3+ lymphocytes were predominantly CD4+ (T-helper marker). Only few CD8+ lymphocytes (cytotoxic T-cell marker) were seen. Sporadically, Leu19+ lymphocytes were found in the portal areas and sinusoids. Lymphocytes did not express appreciable amounts of CD25 (IL-2 receptor) or HLA-DR. Kupffer cells showed a marked expression of CD4 and CD25. These cells belong to the monocytic–histiocytic lineage, and are reported to express CD4 (Poppema & de Ley, 1985) and, after IL-2 stimulation, CD25 (Hancock *et al.*, 1987). In summary, microscopic examination of the liver during rIL-2 induced hyperbilirubinaemia shows features of acute multifocal hepatitis with necrosis and acute pericholangitis. We assume that this represents a non-specific toxic event. In view of the fact that hyperbilirubinaemia does not necessarily recur during subsequent rIL-2 cycles, we suggest that hyperbilirubinaemia does not preclude further treatment with rIL-2 at full dose.

Yours etc.,

C.J.A. Punt*, S.C. Henzen-Logmans,
R.L.H. Bolhuis & G. Stoter,
Dr Daniël den Hoed Cancer Centre,
Groene Hilledijk 301,
3075 EA Rotterdam,
The Netherlands.

*Present address: St Radboud University Hospital, Dept of Medical Oncology, PO Box 9101, 6500 HB Nijmegen, The Netherlands.

### References

HANCOCK, W.W., MULLER, W.A. & COTRAN, R.S. (1987). Interleukin-2 receptors are expressed by alveolar macrophages during pulmonary sarcoidosis and are inducible by lumphokine treatment of normal human lung macrophages, blood monocytes, and monocyte cell lines. *J. Immunol.*, **138**, 185.

MATORY, Y.L., CHANG, A.E., LIPFORD, E.H. *et al.* (1985). Toxicity of recombinant human interleukin-2 in rats following intravenous infusion. *J. Biol. Resp. Modif.*, **4**, 377.

POPPEMA, S. & DE LEY, L. (1985). Unexpected specificity of monoclonal antibodies. In *Peptides of the Biological Fluids, Proceedings Colloquium*, Peters, E.M. (ed.) p. 435.

WEST, W.H., TAUER, K.W., YANELLI, J.R. *et al.* (1987). Constant infusion recombinant interleukin-2 in adoptive immunotherapy of advanced cancer. *N. Engl. J. Med.*, **316**, 898.